# Translation of pharmaceutical 3D printing to clinical point-of-care and industrial manufacturing

**DOI:** 10.1007/s13346-026-02059-z

**Published:** 2026-02-05

**Authors:** Christos I. Gioumouxouzis, Georgios K. Eleftheriadis, Athanasios S. Kyriakidis, Christina Karavasili

**Affiliations:** 1 Laboratory of Pharmaceutical Technology, Department of Pharmaceutical Sciences, Aristotle University of Thessaloniki, 54124 Thessaloniki, Greece; 2 Pharmacare Premium Limited, R&D Department, HHF003 Hal Far Industrial Estate, Birzebbuga, BBG3000 Malta

**Keywords:** 3D printing, Personalization, Industry, Clinical trials, Community pharmacy

## Abstract

Three-dimensional printing of medicines is moving from feasibility to practice across hospital point-of-care manufacture, community-pharmacy compounding and industrial production. Recent signals include a point-of-care printed oral solid dosage form that met bioequivalence in healthy adults, automated capsule preparation with embedded checks in pharmacies and the first approved industrial product. These advances suggest that 3D printing can deliver clinically acceptable quality when responsibilities, verification and documentation are in place. This review integrates evidence across all three settings and offers a critical appraisal of what is required for safe adoption. We examine how regulatory responsibilities should be allocated across distributed sites, how non-destructive testing and chemometric models can be validated for small batches and which digital systems are essential for traceability and oversight. We analyse where economics break even compared with conventional compounding and identify use cases where 3D printing is comparatively advantaged, including low-dose titration, paediatric formats and rapid design iteration. We also outline risks that must be managed, including training and competency, cleaning validation, cross-contamination control and pharmacovigilance across networks. Finally, we propose a near-term agenda that includes standardised conduct of point-of-care trials, multi-site cost and quality benchmarking, explicit guidance on recalls and labelling and deeper industrial–clinical partnerships to turn pilots into routine practice.

**Graphical abstract:**

**Figure:**
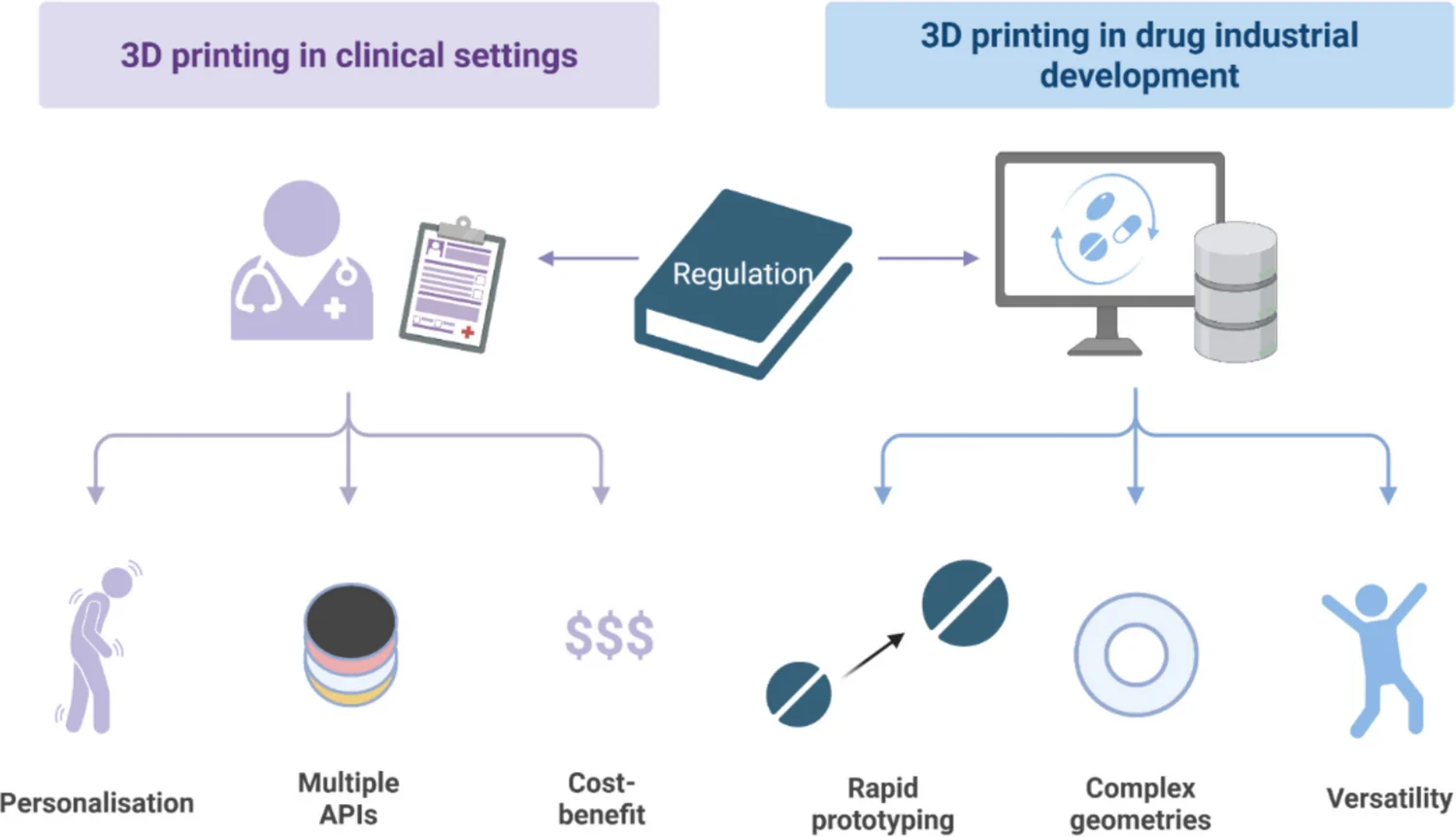

## Introduction

Three-dimensional printing (3DP) of medicines has progressed from proof-of-concept formulations toward a genuine translational phase that spans point-of-care (PoC) manufacture in hospitals, community-pharmacy compounding and automation and industrial production. At its core, 3DP of pharmaceuticals refers to additive manufacturing methods that build dosage forms layer by layer from a digital design. The approaches most often used in pharmacy and industry are semi-solid extrusion [1], fused deposition modelling of drug-loaded filaments [2] and binder jetting of powders [3]. These platforms allow tight control over dose, shape, and internal geometry and can position drug and excipients in specific regions of a tablet to influence disintegration, release kinetics and palatability [4]. They are well suited to small batches and rapid iteration, including patient-specific strengths and paediatric formats that are difficult to source through conventional supply. Constraints, however, remain around printable materials, feedstock properties, in-process controls and the validation of quality attributes for small batches and within these boundaries, 3D printing is best viewed as a complement to tableting and traditional compounding. This convergence is being accelerated by a new regulatory environment, the United Kingdom’s 2025 framework for Modular/Decentralised Manufacture (DM) and PoC medicines, alongside a growing body of clinical, operational and quality-assurance evidence generated in real healthcare settings. Together, these developments suggest that 3DP is shifting from a technology-led curiosity to a regulated, economically defensible modality for delivering personalized therapies [5, 6].

Within the UK regime, decentralised manufacture is organised as a hub-and-spoke model centred on a licensed Control Site that governs quality systems and maintains a Decentralised Manufacture Master File (DMMF) covering authorised PoC production sites. PoC sites operate under the DMMF with defined scopes, controls and oversight. This hub-and-spoke structure clarifies accountability for product quality and pharmacovigilance across distributed locations. By clarifying responsibilities for development, validation, release and ongoing oversight, this framework provides a practical legal pathway for small-batch and patient-specific production close to the point of treatment. The explicit delineation of control, documentation and site authorisation is likely to influence international approaches to distributed and PoC drug manufacture, providing healthcare providers a reference model for safely adopting 3DP [5]. Outside the UK, analogous responsibilities would map to existing GMP and marketing/clinical authorisation structures. The key requirement is explicit allocation of development, validation, release and post-market surveillance across sites.

Signs of clinical readiness are increasingly visible in hospitals. A point-of-care (PoC) printed oral solid has demonstrated pharmacokinetic bioequivalence to its reference in healthy adults, indicating that hospital-level development, manufacture and release testing can yield products suitable for clinical evaluation and, ultimately, routine care. In parallel, methodological guidance is beginning to codify how PoC 3DP trials should be designed and executed within existing regulatory frameworks [7, 8]. In community settings, 3DP is being evaluated not only for personalisation but also for workflow automation and integrated in-process control. Early deployments show that automated capsule preparation with embedded checks can reduce manual handling and standardise compounding steps, with preliminary indications of time and cost efficiencies for low-dose products [9]. These operational signals suggest that 3DP can complement contemporary pharmacy compounding. From an industrial perspective, the field has a clear regulatory precedent in Spritam® (levetiracetam), the first FDA-approved 3D-printed drug, demonstrating that additively manufactured tablets can satisfy stringent quality, safety, and efficacy requirements [10]. Current translational efforts increasingly emphasise dosage-form architectures, process controls, and digital traceability that enable modularity across settings, highlighting the need for harmonised quality systems along the factory-to-bedside continuum.

This review therefore synthesises the state of translation across hospital PoC, community-pharmacy and industrial settings, anchored in the 2025 UK DM/PoC framework and the most recent clinical, analytical and operational evidence. This integrated perspective is distinct from earlier reviews that focused on single settings. Here, the translational continuum is considered as a whole, linking hospital PoC manufacture, community-pharmacy implementation and industrial pipelines to regulatory roles, quality assurance and economics. Consideration is given to regulatory pathways and assignment of responsibility, to quality assurance and non-destructive verification and to economic and workforce impacts. Integrating these domains provides a coherent basis for translation on where 3DP demonstrably adds value today, what barriers remain and how stakeholders can progress toward a safe, economical and scalable deployment.

It must be noted that the present review was written as s thorough critical commentary and situational report and not as a systematic review, due to the limitations by the relatively limited number of relevant published studies in the field. The selection of the reported studies was performed by searching Scopus and Pubmed databases, using [“3D printing” OR “additive manufacturing”] AND [“medication” OR “dosage forms”] AND [“clinical” OR “hospital” OR “community pharmacy”] as keywords. Results were retrieved and after removal of duplicates and irrelevant studies the final 10 (clinical) and 1 (community) studies were selected. No time frame was used, since 3D printing is a relatively new technique.

## Applications and studies in clinical settings

A major goal in the proliferation of pharmaceutical 3D printing is the ability to provide decentralization combined with quality assurance. Taking this into consideration, the UK’s Medicine and Healthcare products Regulatory Agency (MHRA), issued recently its Guidelines on Decentralised Manufacturing of Medicines, providing the framework for the production of medications at the point of care [5]. This collection of documents includes guidance on designation, Marketing Authorization, Clinical Trial Authorisation (and GCP), GMP and pharmacovigilance for decentralized manufacturing.

Beyond the regulatory framework, hospitals need a minimum readiness set before printing for patients. This includes validated equipment (IQ/OQ/PQ), qualified “digital recipes” with version control, trained operators with competency checks and access to non-destructive verification for small batches. A documented change-control process is essential for feedstock lots, printer heads and process parameters, otherwise batch-to-batch drift will undermine clinical reliability.

**Fig. 1:**
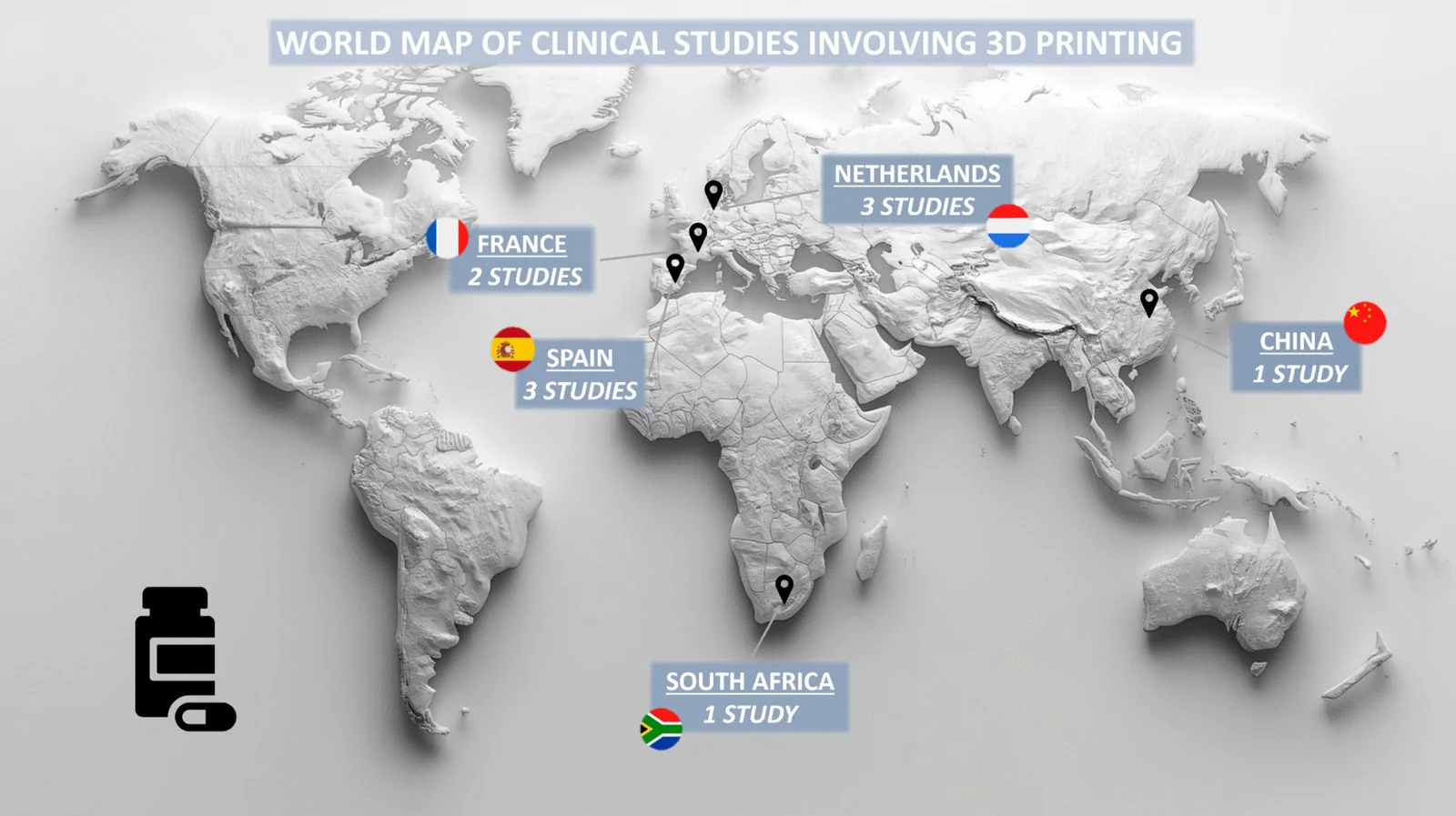
Map depicting the world distribution of 3D printed medications used in clinical trials or 3D printed medications created as part of an ongoing or future clinical study

**Fig. 2:**
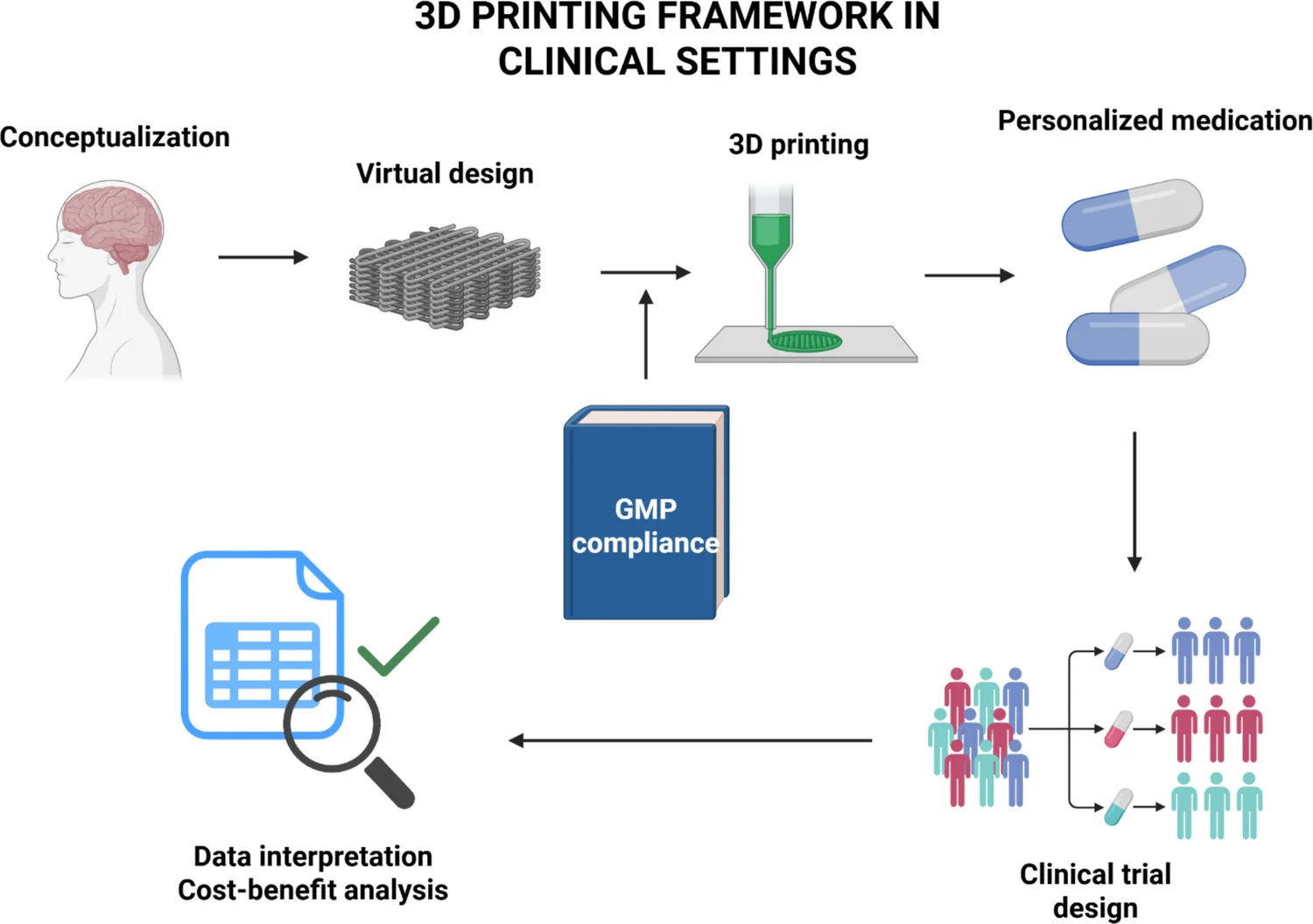
Visualization of 3D printing framework in clinical settings

Prior to the aforementioned regulatory initiative, various attempts were made in order to assess the feasibility and efficacy of introducing 3D printed medicines to clinical practice. With the use of the term “clinical setting studies” we define studies that include: 1) clinical testing of 3D printed dosage forms in healthy subjects or patients or 2) the preparation of 3D printed medications that are intended to be used in such settings or 3) procedures that incorporate the use of 3D printers for the preparation of medication in clinical settings. Figure 1 illustrates the distribution of these attempts worldwide, whereas Fig. 2 summarizes the general pathway of the followed procedure. The first such attempt of conducting a clinical assessment of 3D dosage forms took place in 2019 at Hospital Clínico Universitario de Santiago de Compostela, Spain. In this study, four paediatric patients diagnosed with a rare metabolic disorder (Maple Syrup Urine Disease (MSUD)) and requiring oral supplementation with isoleucine and valine were employed. To conduct the study the researchers prepared both conventional capsules by ordinary manual compounding and personalized chewable formulations by automated 3D printing. More specifically, six types of chewable printlets of different flavours and colours were provided to each patient every two weeks, in the arm of the study that employed the 3D printed formulations. The aim of the study was to evaluate the mean plasma levels of the amino acids after six months of treatment with both types of formulations, as also the acceptability of the 3D printed formulations. The results showed that not only the 3D printed formulations were more acceptable than the conventional dosage forms in terms of palatability and appearance, but also showed mean isoleucine plasma levels closer to the target value and with less variability in comparison to the conventional ones. It must be noted that in this first attempt, 3D printed medications were created using a commercial 3D gummy candy printer and not a GMP-compliant pharmaceutical 3D printer, with profound drawbacks regarding the potential quality of the final products, especially if this approach is to be implemented on a much bigger scale [11]. Results generated on non-GMP food-grade equipment should be treated as feasibility demonstrations rather than evidence for clinical adoption. They help identify acceptability signals and dosing concepts, but product quality and documentation from such platforms are not transferable to regulated workflows without full re-development.

A more recent approach of the same research group took place in the same hospital and was published in 2024. In this study the GMP-compliant pharmaceutical 3D printer M3DIMAKER™ was employed in order to create dosage forms for the treatment of three rare metabolic disorders, Maple Syrup Urine Disease (MSUD), Short-chain Enoyl-CoA Hydratase (ECHS1) deficiency and Ornithine Transcarbamylase (OTC) deficiency. The dosage forms contained nutritional supplementation with the relevant amino acids for each condition: isoleucine and valine in MSUD, isoleucine in ECHS1 deficiency, and citrulline in OTC deficiency. It must be noted that some formulations contained a combination of two different amino acids and a specialized healthcare software (M3DIMAKER Studio™) was employed for the precise calculation of the dimensions of the desired dosage forms. Six types of 3D printed chewable formulations based on different flavours and colours were created using the aforementioned solid-state extrusion 3D printer and administered to each patient (n = 6) every 15 days. Conventional formulations (powder or capsules) were also administered to the patients and plasma levels of the amino acids, as also the acceptability of the formulations, were assessed for both 3D printed and conventional dosage forms. The results of the study showed that both chewable 3D printed and conventional medicines effectively maintained amino acid levels within the target levels (small sample size precluded any statistically significant differences). The study also demonstrated that 3D printed formulations were more acceptable than the conventional ones, based on their rating on the hedonic scale by the young patients. Moreover, the study revealed the heterogeneity of the color and taste preferences of the patients, highlighting the advantage of 3D printing in creating personalized dosage forms based on the personal preferences of each individual [12]. It should be mentioned that hedonic scales capture preference, but are sensitive to expectation and novelty effects, especially in paediatric populations. Future studies should pair acceptability with adherence proxies and caregiver burden and should report whether flavour or colour personalisation meaningfully changes persistence with therapy over time.

M3DIMAKER 2 was also employed in the creation of personalized dosage forms for the ongoing OPERA clinical trial aiming at providing medication to women with breast cancer. These patients are scheduled to receive both anticancer drug tamoxifen, as also antidepressants venlafaxine and duloxetine. In this study 3D printer was used as a capsule filler, using modified pellets dispensing printheads to fill capsules with venlafaxine or duloxetine pellets or precise quantity of semisolid pharma-ink containing tamoxifen. The employment of 3D printing resulted in rapid production of the dosage forms (a full batch of 200 units could be manufactured in less than 45 min) with exceptional dosing precision [7]. In this context, printing as a capsule-filling method can be rapid, but safe release still hinges on an agreed sampling plan. A practical approach is to combine in-process controls with non-destructive verification (e.g., NIR/Raman) and reserve destructive testing for method validation and periodic surveillance.

Furthermore, this Semi-Solid Extrusion (SSE) printer was also employed in the manufacturing of personalized chewable hydrocortisone formulations (printlets), intended to be used in an upcoming clinical study at Vall d’Hebron University Hospital in Barcelona, Spain. These formulations will be used for the treatment of adrenal insufficiency of paediatric patients and therefore printlets with a variety of colors and flavors were prepared in order to mask the bitterness of hydrocortisone and evaluate the acceptability of the formulations, since a variety of options is provided. Stability, mass uniformity and acceptable in vitro drug release demonstrated the suitability of the formulations for use in the upcoming clinical trial [13]. It is important to mention that chewable and paediatric dosage forms are often moisture-sensitive and flavour-laden. Real-time stability under intended storage, child-resistant packaging that preserves taste masking and in-use stability (e.g., split packs) should therefore be established before routine use. In addition, flavouring and colouring agents aid acceptability but can introduce allergens or intolerances. Site-level excipient policies and parent information sheets should accompany personalised flavours and substitutions must be captured in the recipe history.

Semi-solid extrusion was also employed in a Chinese study to create Levothyroxine sodium tablets administered to low birth weight (LBW), very low birth weight (ELBW) and very low birth weight (VLBW) premature infants with transient hypothyroxinemia. A total of 91 infants participated in the study at Shunde Women’s and Children’s Hospital (Guangdong Medical University), with 42 receiving 3D printed formulations of Levothyroxine and 49 manually subdivided (via grinding and shearing) Levothyroxine formulations, both categories containing the exact required dose for each infant. The results for LBW infants were similar for the two types of formulations, whereas ELBW and VLBW infants presented both better thyroid hormone concentrations and better clinical outcomes (such as weight gain) in the case of administration of 3D printed formulations, in comparison to manually adjusted ones. This result is a direct consequence of the reported better stability, less weight variability, higher content accuracy and less weight loss of the 3D printed formulations [14]. Differences in hormone levels and weight gain may reflect improved dose accuracy and handling losses rather than an intrinsic advantage of the 3D printed dosage form. Since, non-randomised designs, co-interventions and NICU practice variability can confound outcomes, replication with randomisation or robust adjustment would significantly strengthen the claim.

Another study employing SSE for the production of 3D printed dosage forms was conducted at Leiden University Medical Center (LUMC) in 2022. In this study 3D printed sildenafil formulations (usually used to treat pulmonary arterial hypertension in young children) were created via an SSE 3D printer and administered to 12 healthy adult volunteers in a randomized, open-label, 2-period, crossover, single-dose study, comparing their pharmacokinetic profile to the commercially available sildenafil tablets Revatio™. The comparison between the 90% confidence interval (CI) of the geometric mean ratio (GMR) of test to reference treatments of AUC_0-t_ and C_max_ revealed the bioequivalence of the 3D printed and commercial formulations, establishing the feasibility of creating a point-of-care 3D printed formulation bioequivalent to the marketed ones. The study emphasizes in the establishment of reasoning bioavailability of 3D printed formulations, as it is not feasible to assess personalized medicines prior to use since they are manufactured for specific patients with varying doses and release profiles [8]. Demonstrating bioequivalence in healthy adults is a major step for PoC printing. Translationally, the next steps should be paediatric-appropriate dose strengths, palatable matrices and bridging of PK, where ethical and feasible, coupled with pragmatic endpoints such as dose titration accuracy.

An older study employed Powder Bed Printing (PBP) in which binding liquid is sprayed on layers of powder to create 3D objects. Pseudoephedrine hydrochloride (PEH) was used as the model drug and three cubic shaped dosage forms with core–shell structure were created. The difference of the three formulations was the different amount of Kollidon™ and hydroxypropylmethyl cellulose (HPMC) used in the powder matrix, resulting in formulations with different release duration (8 h, 12 h and 16 h). The structure facilitated the near zero-order controlled-release of the active ingredient, via a diffusion mechanism. The in vivo performance of the formulations was evaluated using a single-dose, randomized, open-label, four-way crossover clinical study composed of 10 healthy volunteers. The dosage forms (administered after being loaded in capsules) demonstrated excellent in vitro*/*in vivo correlation for a prolonged time period, exhibiting the potential of 3D printing for rapid prototyping of controlled-release formulations [15].

Palatability of 3D printed formulations was evaluated in another study, where chewable printlets containing a combination of Sulfamethoxazole and Trimethoprim, were 3D printed from pharma inks manufactured in the pharmacy of Gustave Roussy hospital in France. Double-layered printlets were produced with acceptable accuracy and speed and the preliminary taste testing in 6 healthy volunteers revealed a significant reduction of persistent bitterness, in comparison to the commercially available suspension containing the combination of the 2 API’s [16].

Recently, Hospital Pharmacy of Hamburg University Medical Centre Hamburg-Eppendorf reported its attempt to incorporate 3D printing into the procedures of their significantly automated and digitalized environment. For this reason, a suitable drug combination (levodopa/carbidopa) was selected as model drug and a relevant IT concept was developed and adapted to the hospital’s current IT infrastructure. The next steps of the project include the manufacturing of a suitable formulation via 3D printing, according to the personalized needs of the inpatients, which will be monitored with smart wearable devices. The significance of this project lies at the importance of incorporating 3D printing procedure into the digital and automated environment of modern hospital pharmacies [17].

A significant question regarding 3D printed medications in clinical settings are the costs of manufacturing and cost-effectiveness of the whole procedure. A recent study from the Department of Hospital Pharmacy of Erasmus University Medical Center tried to evaluate these costs using an FDM (Fused Deposition Modelling) 3D-printed hydrocortisone formulation (M3DICORT). The study reached the conclusion that the cost of a single formulation could vary between €1.58 and €3.11 (lowest estimates represent the best-case scenario and production scale up and the highest represent worst-case scenario and the production of small quantities). It must be noted that costs may reduce if a third party provide standardized pharma-inks or coils of filament to feed 3D printers at clinical settings. The above highlight the potential feasibility of producing relatively cost-effective medications for personalized treatment [18]. Another study from the same group of researchers estimated the cost of SSE 3D printed immediate and sustained release hydrocortisone tablets at < € 3.00 per tablet for both release profiles, indicating that both 3D printing methods result in a comparable manufacturing cost in clinical settings [19].

Nevertheless, not all hospital or investigational needs justify use of 3DP. For stable, high-dose, single-strength products with long shelf-life and large predictable volumes, conventional industrial supply or standard pharmacy compounding will remain more efficient. Likewise, indications requiring intensive stability testing (e.g., narrow therapeutic index drugs) may exceed the validation effort feasible for small PoC batches. A practical adoption test is whether 3DP reduces time-to-patient, total cost of quality, or medication risk versus the incumbent route. If none improve, 3DP may not be the optimal pathway to pursue.

A recent review summarizes the challenges in clinical applications of 3D printed medications and the associated clinical trials. Several tests should be performed regrading mass and content uniformity, friability, in vitro disintegration and dissolution, stability for the desired time period and microbiological quality. Patients involved should be recruited using a robust methodology to ensure representative results, corresponding to diverse populations. Moreover, certified and validated equipment should be used according to the existing or the upcoming regulatory framework to ensure compliance with the necessary standards [20].

Table 1 summarizes the basic characteristics of the aforementioned attempts to introduce 3D printing into clinical practice.

**Table 1:** Summary of studies employing pharmaceutical 3D printing in clinical settings

Setting (site/country)	Publication year	No of participants/design	Disease	API	Printing process	Primary endpoint	Outcome	Key limitations	Ref
Hospital Clínico Universitario de Santiago de Compostela, Spain	2019	4/Single-centre, pilot, prospective, crossover	Maple Syrup Urine Disease (MSUD)	Isoleucine/valine	SSE	Plasma concentration, Acceptability	Positive	Nr of participants, Non GMP equipment	[11]
Hospital Clínico Universitario de Santiago de Compostela, Spain	2024	6/Prospective, single-centre, crossover, observational	Maple Syrup Urine Disease (MSUD), Short-chain Enoyl-CoA Hydratase (ECHS1) deficiency, Ornithine Transcarbamylase (OTC) deficiency	Isoleucine/valine/citrulline	SSE	Plasma concentration, Acceptability	Positive	Nr of participants	[12]
Cancer Hospital Gustave Roussy, Paris, France	2024	OPERA trial -ongoing	Breast cancer	Tamoxifen/venlafaxine/duloxetine	SSE/Capsule filling	N/D	-	-	[7]
Vall d’Hebron University Hospital in Barcelona, Spain	2024	Upcoming	Adrenal insufficiency	Hydrocortisone	SSE	Plasma concentration, acceptability	-	-	[13]
Shunde Women’s and Children’s Hospital (Guangdong Medical University)	2023	91/Prospective, single-centre	Transient hypothyroxinemia	Levothyroxine sodium	SSE	Plasma concentration, weight gain	Positive	No crossover study design	[14]
Leiden University Medical Center (LUMC)	2023	12/Randomized, open-label, 2-period, crossover, single-dose	Pulmonary arterial hypertension	Sildenafil	SSE	PK/BE	Positive	Nr of participants, Healthy adult volunteers	[8]
Biopharmaceutics Research Institute, Rhodes University, Grahamstown, Republic of South Africa	2006	10/Single-dose, randomized, open-label, four-way crossover	-	Pseudoephedrine hydrochloride (PEH)	Powder Bed Printing (PBP)	Zero-order controlled-release, In vitro*/*in vivo correlation	Positive	Nr of participants, Healthy adult volunteers	[15]
Cancer Hospital Gustave Roussy, Paris, France	2025	6/Unblinded, prospective, single-centre	Infection	Sulfamethoxazole/Trimethoprim	SSE	Acceptability	Positive	Nr of participants	[16]
Hospital Pharmacy of Erasmus University Medical Center	2025	-	Adrenal insufficiency	Hydrocortisone	FDM	Cost-effectiveness	Positive	Not currently used in clinical trial	[18]
Hospital Pharmacy of Erasmus University Medical Center	2025	-	Adrenal insufficiency	Hydrocortisone	SSE	Cost-effectiveness	Positive	Not currently used in clinical trial	[19]

*PK/BE* Pharmacokinetics/Bioequivalence, *N/D* Not determined, *SSE* Solid State Extrusion, *FDM* Fused Deposition Modelling, *PBP* Powder Bed printing

Concluding, regarding clinical application of 3D printing, evidence to date remains limited. Most studies are single-centre, enrol small cohorts and have short follow-up, with outcomes often restricted to surrogate endpoints. External validity is therefore uncertain. Operator-dependent variability is a further constraint in point-of-care settings; sustained performance requires documented SOPs, periodic competency assessment and proficiency checks. Pharmacovigilance must be planned across sites in advance, with clear assignment of responsibilities for signal detection, case processing, field safety notices and product recalls, and with routine testing of these procedures.

Economic findings are encouraging but context dependent. Unit costs should be derived through micro-costing that separates materials, labour, equipment depreciation and maintenance, and the share of quality-assurance overhead, while accounting for throughput, yield, and equipment utilisation. Estimates are sensitive to staff grade, local licensing requirements and the intensity of QA sampling. Current case studies indicate that for low-dose products requiring frequent titration, small-batch 3D printing can approach and in some scenarios match, the cost of conventional compounding, particularly when automation reduces manual handling and rework. Nevertheless, since initial studies revealed encouraging results in terms of acceptability, bioavailability and overall feasibility, clinical studies regrading 3D printed formulations in hospital settings are expected to multiply, taking into consideration the numerous advantages (personalization, dose accuracy, multi-drug dosage forms etc.) of these formulations.

Despite encouraging early signals, the current body of clinical evidence for pharmaceutical 3D printing remains limited in scope and maturity. Most reported studies are single-centre investigations with small cohorts and short follow-up periods, frequently relying on surrogate endpoints such as pharmacokinetic equivalence, plasma concentration stability or acceptability scores. While these outcomes are appropriate for feasibility assessment, they do not yet establish long-term clinical benefit, robustness of performance across operators and sites or sustainability of quality over extended use.

A further limitation is the heterogeneity of printing platforms, materials and quality control strategies used across studies, which complicates cross-study comparison and generalisation. In several early investigations, non-GMP or food-grade equipment was employed, meaning that results should be interpreted as proof-of-concept rather than directly transferable to regulated clinical workflows. Even in GMP-compliant environments, point-of-care manufacture introduces operator-dependent variability and necessitates clearly defined release strategies, including the use of validated non-destructive analytical methods for small batches.

From a translational perspective, future clinical studies should move beyond feasibility and bioequivalence to address reproducibility across sites, robustness of digital recipes and integration of pharmacovigilance and recall procedures within decentralised networks. Standardisation of trial conduct, documentation and quality assurance will be essential to enable meaningful comparison between 3D-printed and conventionally prepared medicines and to support wider clinical adoption.

## Applications and studies in community pharmacies

3D printing in community pharmacies comprises a more complicated issue, as restrictions inherent to the reality of community pharmacy render practical applications of pharmaceutical 3D printing more difficult to implement. Nevertheless, a recent paper explores the use of a 3D printer with specially modified printheads that have the ability to precisely fill capsules for the production of small batches of galenic formulations [9]. More specifically, semi-solid extrusion printers were used for the automated filling of capsules or blisters, while incorporating an integrated quality control system. The minoxidil capsules prepared, met the European Pharmacopeia quality standards and were stable for 3 months after compounding. The preparation was conducted in a Spanish community pharmacy with a compounding license and a conditioned laboratory, after installing the 3D printer and performing the necessary Qualification (Installation, Operational and Performance) by the printer’s manufacturer. 870 minoxidil capsules were prepared in total and administered to 9 patients. Automated production of capsules via 3D printing reduced the total time needed by approximately 10%, the time of manual labour by approx. 55% and also incorporated an automated quality control analysis (in comparison to the conventional method). Moreover, the short-term total cost of the preparation via 3D printer was reduced by 20–35% (mainly due to the less involvement of the pharmacist). Additional advantages of the automated preparation include the limited exposure of the operator to the API’s and the reduction of the manual stress of the operator.

Summarizing, in community pharmacies, preliminary data indicate reduced manual labour and short-term total cost reductions with automated 3DP capsule preparation. Formal micro-costing across multiple pharmacies and sensitivity analyses (labour rates, batch sizes, QA sampling frequency) are needed to define robust break-even thresholds. While reduced manual handling is beneficial, cleaning validation remains critical to prevent cross-contamination across products. Documentation of cleaning procedures, residue limits and changeover verification should be adapted to the printer design and the APIs handled. Taking into consideration the above, capsule filling can be an attractive field of implementation of 3D printing in community pharmacy settings, under certain circumstances.

The production of personalised 3D printed dosage forms in community pharmacies remains extremely challenging, as it requires expertise and equipment that possibly exceeds their capabilities. An approach that will simplify the procedures could be the supply of industrial API-prefilled cartridges for SSE printing or coils of filament for FDM printing as previously discussed [21].

Evidence for the implementation of pharmaceutical 3D printing in community pharmacy settings is currently extremely limited, with only a single published study reporting real-world application. While the reported reductions in manual labour, operator exposure and short-term costs are promising, conclusions must be interpreted cautiously due to the absence of comparative multi-site data and long-term economic evaluation.

Importantly, community pharmacy environments pose distinct translational challenges compared with hospital PoC settings, including constraints related to space, staffing, training, quality oversight and cleaning validation in multi-product workflows. Although automation via 3D printing may reduce manual handling, it does not eliminate the need for rigorous documentation, validated cleaning procedures and defined responsibility for quality release. These requirements may exceed the operational capacity of many community pharmacies, particularly for personalised dosage-form printing rather than automated capsule filling.

From a translational standpoint, 3D printing in community pharmacies is therefore more likely to be viable initially for narrowly defined applications, such as automated preparation of standardised, low-dose formulations using pre-qualified feedstocks supplied by industry. Broader implementation of fully personalised 3D-printed medicines in this setting will require further regulatory clarity, dedicated training frameworks and robust economic justification supported by multi-centre studies.

## Translation of 3D printing into industrial pharmaceutical products

With the use of the term “industrial setting”, we refer to the use of the 3D printing for the mass production of dosage forms from companies, designated for commercial use. Application of 3D printing in order to create pharmaceuticals in an industrial scale is presumably the most challenging but also the most promising field of translation, due to the fact that it enables the creation of really complex dosage forms on a large scale. A landmark in industrial translation was the approval of Aprecia’s SPRITAM® from FDA. SPRITAM® (containing the antiepileptic levetiracetam) was licensed in 2015 [10] for the treatment of partial-onset seizures, myoclonic seizures, juvenile myoclonic epilepsy, primary generalized tonic–clonic seizures and certain types of generalized epilepsy. SPRITAM® is manufactured using a subcategory of 3D printing called Drop-on-Solid or Powder Bed Printing in which the creation of 3D objects is achieved via spraying of binding liquid on layers of powder. The subsequent evaporation of the binding liquid results in the creation of loosely bound objects, which in the case of SPRITAM® is an advantage, since this dosage form is an orodispersible tablet, intended for rapid disintegration in the mouth using the patient’s saliva [22]. The approval of SPRITAM® is considered a landmark, as it first demonstrated the feasibility of using 3D printers for the industrial production of pharmaceuticals. Recently Aprecia’s Z-FORM 3D printing platform technology, along with machine learning-based process analytical technologies (PAT), allowed for precise layer-by-layer formation of customizable pharmaceutical drug products produced directly in their primary packaging [23].

The initial success of Spritam® was followed by a 5-year gap in industrial applications of 3D printed dosage forms. Nevertheless, during the current decade two companies (one based in the US and the other in China) have made significant steps in manufacturing 3D printed medicaments. The first one is NY based Laxxon Medical (originally founded in 2017 and retaining its research and development facilities in Jena, Germany) using its proprietary technology called SPID® (Screen Printed Innovative Drug) which is a form of 3D screen printing technology. The working principle of this technology is the spreading of the printing material on a flat surface and the subsequent squeezing of the material through orifices of the desired shape, followed by curing of each layer (in order to solidify it) before the deposition of the next [24]. Laxxon claims that it has the capability to print 1.5 M dosage forms per day without the need to change the manufacturing process. It has currently 13 drugs in its internal pipeline in various stages of development (Formulation & Product Development, Pre-Clinical, Clinical & Registration (based on bioequivalence studies)), aiming at the treatment of a wide spectrum of diseases (Epilepsy, Diabetes/Metabolic Syndrome, Rheumatoid Arthritis, Parkinson's Disease, Renal Cell Carcinoma, Thromboembolism, Prostate Carcinoma, Attention-Deficit/Hyperactivity Disorder etc.) [25].

The other major company actively engaging in industrial pharmaceutical 3D printing is the Chinese Triastek founded in 2015. Their approach exploits the potential of Melt Extrusion Deposition (MED^@^) alone or coupled with Micro-Injection Molding (MIM) or Semi-Solid Extrusion (SSE). MED^@^ converts powder feedstock into molten mixtures, subsequently deposited on a surface layer by layer. The process is solvent-free, it does not require the intermediate creation of feeding filaments and it does not involve any post processing. The manufacturing of tablets is followed by Linear Laser Scanning to determine the size of each tablet in real time. Triastek claims ability of manufacturing up to 50 M tablets per year [26]. Until now they claim to have developed more than 70 novel dosage form designs, creating microstructures aiming at solubility enhancement, gastric retention, intestine targeting, modified release or administration of oral biologics [27]. In 2025 they were granted their first FDA Investigational New Drug approval for a proprietary 3D-printed product for the treatment of rheumatoid arthritis (RA) [28] and since then they have 4 products (against Pulmonary Arterial Hypertension, Atrial Fibrillation/Venous Thromboembolism, Insomnia and IgA nephropathy) in the clinical or preclinical pipeline [29]. Moreover, in 2023 Triastek announced a partnership with Boehringer Ingelheim, aiming at the rapid creation of different formulation prototypes that can be used to examine the behavior of a variety of formulation and determine the best suited for further development and mass production (a process called 3D printing Formulation by Design (3DFbD®)). This approach can significantly accelerate the development and increasing the success rate of new dosage forms [30]. Also, in 2024 Triastek announced its collaboration with BioNTech for the development of sophisticated 3D printed platforms aiming at delivering RNA therapeutics at specific sites of the gastrointestinal tract, in order to minimize potential degradation of the sensitive biologics [31].

The industrial applications of 3D printing technology in pharmaceutical manufacturing present transformative opportunities and concepts for personalized medicine and improved patient outcomes. However, addressing major regulatory and GMP considerations is required for broader adoption of this technology in industrial settings.

### Regulatory considerations

The regulatory landscape for industrial 3D printing of pharmaceuticals, is currently in a state of active development. Major regulatory agencies worldwide are working to establish comprehensive frameworks that balance innovation with patient safety. While traditional pharmaceutical regulations form the foundation, new guidelines are emerging to address the unique challenges posed by additive manufacturing technologies. With the approval of Spritam®, the FDA takes a risk-based approach to 3D-printed pharmaceuticals, treating them under existing drug approval pathways [32, 33]. On the other hand, the FDA's Framework for Regulatory Advanced Manufacturing Evaluation (FRAME) initiative represents the most comprehensive regulatory approach currently under development [34]. The aim of FRAME is to establish a regulatory framework that facilitates the adoption of advanced manufacturing technologies, ultimately delivering benefits to patients. FRAME focuses on four key advanced manufacturing technologies, i.e., end-to-end continuous manufacturing, distributed manufacturing, distributed manufacturing units at non-traditional host sites, and artificial intelligence. Through this initiative, the FDA has published discussion papers on distributed manufacturing and artificial intelligence in drug manufacturing, seeking stakeholder feedback to inform future policy development.

The European Medicines Agency (EMA) has included 3D printing and 3D bioprinting in its revised list of enabling technologies [35]. Along the same lines as FDA’s approach, 3D-printed pharmaceuticals can in principle be approved within the EU with existing drug approval pathways. 3D-printing can be considered as a non-standard process under EMA according to Annex II to Note for Guidance on Process Validation [36]. Within this framework, the case may be that the manufacturing process has either not been previously approved for a pharmaceutical product in the EU, or when previously approved, it is categorized as an established process known to be complex. Such a categorization requires a more robust approach during process validation, excluding in certain cases the possibility of using pilot data to support a new Marketing Authorisation application [37].

Based on the above, it may be concluded that the EU regulatory framework remains technologically neutral; meaning manufacturers must ensure compliance with existing machinery directives and medical device regulations [38]. At the same time, under the EU Medical Device Regulation (MDR), 3D printing activities that may be carried out within healthcare institutions are covered by the “Health Institute Exemption” (Article 5). This exemption requires the implementation of robust quality management systems, adherence to all General Safety and Performance Requirements and maintenance of thorough documentation [39].

### GMP considerations

Since the regulatory landscape for 3D printed pharmaceuticals operates within the existing Good Manufacturing Practice (GMP) frameworks, the industrial application presents unique challenges that require specialized consideration. The current regulatory framework classifies 3D-printed drug products as pharmaceuticals, requiring compliance with established cGMP standards — specifically 21 CFR Parts 210 and 211 in the United States and EudraLex Volume 4 in the European Union. These products must also comply with established Chemistry, Manufacturing and Control (CMC) requirements specified in 21 CFR 200 s and 300 s, as well as other applicable guidance for 505(b)(1) and 505(b)(2) submissions for USA; the EMA Scientific Guidelines provide equivalent requirements for EU [40]. This ensures that, despite the innovative production method, core quality and safety standards for pharmaceutical manufacturing are maintained. Interestingly, the UK has a dedicated legal framework that explicitly enables PoC/DM manufacturing models for medicines, including 3D‑printed medicinal products, under the Human Medicines (Amendment) (Modular Manufacture and Point of Care) Regulations 2025 and associated MHRA guidance. This framework came into force in July 2025 and amends the Human Medicines Regulations 2012(a) to create a specific regime for medicines manufactured at or near the site of care. In this context, it covers PoC manufacture in hospitals, clinics, mobile units and certain community or near‑home settings, provided manufacture is not on a large scale or by an industrial process. It is intended for highly personalized or short‑shelf‑life medicines that cannot practically be supplied via traditional, centralized manufacturing and distribution routes. Although tailored to decentralized settings, this framework still mandates that medicines manufactured under PoC/DM settings, including 3D‑printed products, must meet the usual requirements for quality, safety and efficacy and operate under a modified GMP‑based system. Similarly, the FDA’s ongoing (FRAME) initiative is actively exploring ways to address the unique regulatory challenges posed by distributed manufacturing models. There are specific exemptions for compounded human drug products that apply to section 501(a)(2)(B) on cGMP requirements, section 502(f)(1) on labeling requirements, and section 505 on new drug application approvals under the Food, Drug, and Cosmetic Act. Although not directly linked to 3D printing, this framework sets general exemptions for qualifying compounded drugs. However, these exemptions are subject to strict conditions and do not remove the obligation to ensure robust quality control and patient safety [20, 41, 42].

The successful integration of GMP-compliant 3D printing into pharmaceutical manufacturing depends on the availability of specialized equipment, which is designed to meet stringent regulatory standards. A major challenge to the broader adoption of this technology has been the limited availability of 3D printers that comply with cGMP requirements. Towards this step, FabRx’s M3DIMAKER series and the LUMC Hospital Pharmacy's MedPrint system marked significant milestones, by providing GMP-certified 3D printing technologies. Since 3D-printed pharmaceuticals must meet the same pharmacopoeial standards as conventionally manufactured drugs, state-of-the-art printers may host sophisticated quality control systems; for example, analytical balances to verify mass uniformity, PAT controls for real-time monitoring with sensors and machine vision for defect detection, and automated process documentation to meet 21 CFR Part 11 requirements for electronic records. Such systems allow continuous oversight and precise control of critical process parameters throughout manufacturing, thus enabling a substantial advancement in GMP compliance of 3D printers [43–48]. Non-destructive PAT tools may include Near-Infrared (NIR) Spectroscopy, which provides the option for real-time analysis of drug content with high accuracy, Raman spectroscopy for molecular identification and quantification, and machine vision systems for AI-driven visual inspection [46, 49, 50].

Accordingly, GMP-compliant 3D printing facilities are required to adhere to strict environmental and operational standards, comparable to those of conventional pharmaceutical manufacturing. Qualification during installation (IQ) and operation (OQ) are essential cGMP requirements, while their performance against a particular process must be studied, documented and validated (PQ). General requirements of EU-GMP, particularly chapters 3, 4 and 5 of Eudralex vol.4, are absolutely necessary. Particular attention, specific to 3D-printing, may be given to personnel training [51] and process validation [52, 53]. Addressing cross-contamination issues and cleaning validation with the novel manufacturing equipment needed for 3D-printing can potentially be the most challenging factor in incorporating such unfamiliar technology into an industrial pharmaceutical setting. The use of dedicated equipment or parts may be the way forward to addressing this issue.

**Fig. 3:**
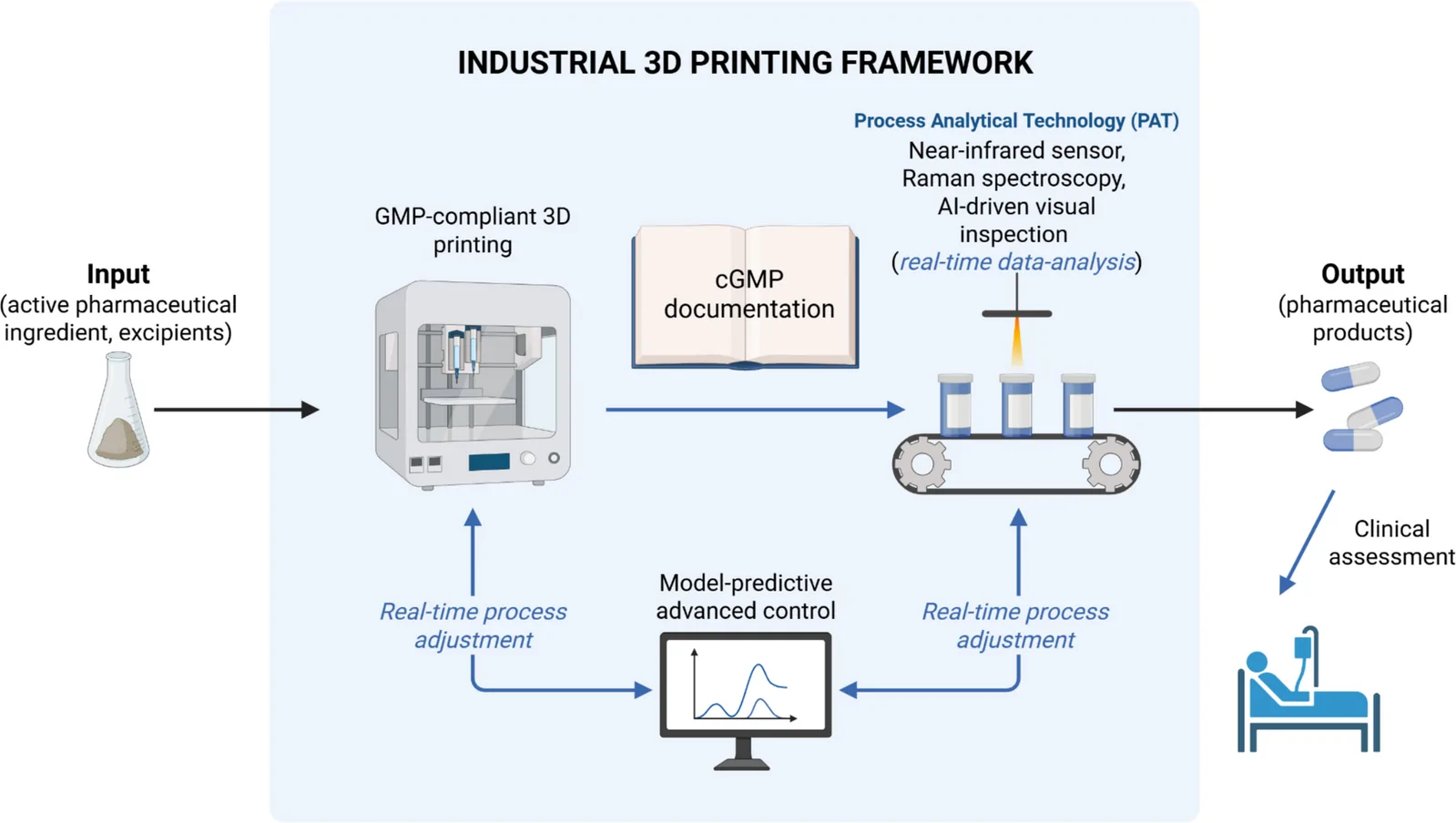
Visualization of 3D printing framework in industrial drug development

The integration of 3D printing into pharmaceutical manufacturing must further align with established Pharmaceutical Quality System (PQS) frameworks, as described in ICH Q10. This involves comprehensive knowledge management across the product lifecycle, application of ICH Q9 quality risk management principles, and continuous improvement processes to ensure compliance and optimization [54, 55]. Moreover, Quality by Design (QbD) is critical for 3D-printed pharmaceuticals. It is necessary to identify Critical Quality Attributes (CQAs) that influence product quality and performance, e.g., content uniformity, hardness and stability, Critical Process Parameters (CPPs) such as printing temperature, speed and material flow and Critical Material Attributes (CMAs), like thermal, mechanical and rheological properties of both active ingredients and excipients. Process validation must demonstrate that the 3D printing process produces products consistently, while meeting the predefined quality and specifications criteria across single and multiple build cycles, as well as between different machines [38, 54]. Standard Operating Procedures (SOPs) should encompass all aspects of production, including traceability of raw materials, manufacturing, printer usage logs, batch release, equipment and facility cleaning and personnel training [20]. Lifecycle Management, as per ICHQ12, should play a crucial role in such products. The industrial framework of 3D printed medications is summarized in Fig. 3.

Nevertheless, industrial viability depends on overall equipment effectiveness, unit cycle time, and yield. High-speed rotary presses already deliver very large volumes at low unit cost once a formulation is established. To compete, industrial 3D printing has potential to create value that conventional processes cannot. Examples include complex spatial drug–excipient architectures with clinically meaningful performance, rapid design iteration that shortens development timelines, and highly personalised strength ladders without retooling. In practice, industrial 3D printing makes the most sense when those distinctive capabilities are required, while conventional tableting remains more efficient for high-volume single-strength products with simple dissolution performance.

Generally, adapting quality systems to small, distributed batches requires validated non-destructive tests, robust chemometric models with independent validation sets and suitability assessments for content/mass uniformity, dissolution, and stability. Cleaning validation and cross-contamination control are also critical in multi-product settings. Release strategies should combine in-process controls with rapid non-destructive verification, reserving destructive tests for method validation and periodic verification. If the above prerequisites are met, industrial pharmaceutical 3D printing can play an important role in the cost-effective manufacturing of dosage forms of extreme complexity, which are impossible or not economically viable to be produced with conventional methods.

## Conclusions

3D printing of medicines has progressed into a translational phase that now spans hospital point-of-care manufacture, community-pharmacy compounding and industrial pipelines. The emergence of decentralized-manufacture frameworks, particularly the UK’s 2025 model, provides a legal and operational basis for controlled small-batch production. Early evidence, ranging from pharmacokinetic bioequivalence of a point-of-care product to automated capsule preparation in pharmacies, to industrial precedents such as Spritam^®^, shows that adoption is technically and economically feasible for selected use cases.

Moving forward, success will depend on adapting quality systems to distributed contexts including validated non-destructive testing, digital batch records, cleaning validation, cross-contamination controls, and clear role allocation between Control Sites and PoC sites. Workforce training and competence frameworks must evolve to support these responsibilities, while economic viability will hinge on streamlined workflows, automation to reduce manual handling, and transparent costing models that account for QA overhead.

Near-term priorities shall include the standardization of PoC clinical trial conduct and release testing, comparative cost/quality benchmarking against conventional compounding, clear regulatory guidance on labelling, recalls, and pharmacovigilance in distributed networks, and strengthened industrial-clinical partnerships. Addressing these will be critical to realizing the promise of safe, economical, and scalable 3D-printed medicines.

## Data Availability

The datasets generated during and/or analysed during the current study are available from the corresponding author on reasonable request.
